# Urethral discharge as an early manifestation of urinary tract infection in children ≤24 months old

**DOI:** 10.3389/fped.2023.1149218

**Published:** 2023-06-19

**Authors:** Nai-Wen Fang, Shih-Hsiang Ou, Yu-Shan Huang, Yee-Hsuan Chiou

**Affiliations:** ^1^ Division of Pediatric Nephrology, Department of Pediatrics, Kaohsiung Veterans General Hospital, Kaohsiung, Taiwan; ^2^ Department of Pediatrics, Pingtung Veterans General Hospital, Pingtung, Taiwan; ^3^ Division of Nephrology, Department of Internal Medicine, Kaohsiung Veterans General Hospital, Kaohsiung, Taiwan

**Keywords:** urinary tract infection, urethral discharge, gross hematuria, malodorous urine, *Klebsiella pneumonia*

## Abstract

**Background:**

Children with febrile urinary tract infections (UTIs) are prone to kidney scarring if they are not treated promptly; however, ambiguous symptoms before fever onset makes the early detection of UTIs difficult. Our study aimed to identify urethral discharge as an early manifestation in children with UTI.

**Methods:**

This study enrolled 678 children younger than 24 months with paired urinalysis and culture performed between 2015 and 2021; 544 children were diagnosed with UTIs. Clinical symptoms, urinalysis, and paired urine culture results were compared.

**Results:**

Urethral discharge was observed in 5.1% of children with UTI and yielded a specificity of 92.5% for diagnosing UTI. Children with urethral discharge had a less severe UTI course, furthermore, nine of them received antibiotics before fever occurred and seven of them were free of fever during UTI course. Urethral discharge was associated with alkalotic urine and *Klebsiella pneumonia* infection.

**Conclusions:**

Urethral discharge is an early symptom in children with UTI; it may present before fever onset and help ensure prompt antibiotic intervention.

## Introduction

Urinary tract infection (UTI) is the most common bacterial infection in children; in children younger than 24 months old, the prevalence of UTI was 4.5%–7.2% in children with fevers (1). Without prompt intervention, children with cystitis may develop acute pyelonephritis (APN), renal abscess, or sepsis; furthermore, APN may lead to kidney scarring (2, 3), which is potentially related to long-term hypertension (4), chronic kidney disease, and end-stage kidney disease (5, 6). In fact, the odds ratio of kidney scarring increases by 0.8% every hour that antibiotic intervention is delayed in children with febrile UTI (7). Therefore, early recognition of UTI in children is crucial to prevent subsequent sequela.

The symptoms of UTI in children younger than 24 months old are ambiguous and may include nausea, vomiting and lethargy (8). Clinically, a fever without an identifiable infection focus may be the sole symptom in children with UTI (9); it is thus difficult to diagnose and treat children in this age group before a fever develops. Two UTI symptoms that can be detected before fever onset are malodorous urine and gross hematuria; however, malodorous urine is somewhat subjective and may be influenced by a child’s urine concentration and hydration status, and gross hematuria may be confused with urate crystal in young infants with dehydration. Both symptoms warrant an examination of the urine, but they are not specific to UTI. In the current study, we identified urethral discharge as a unique symptom of UTI in children before fever onset. Careful observation of this symptom may warrant an urinalysis, which may facilitate early diagnosis and treatment of children with UTI.

## Materials and methods

### Enrollment of children

From January, 2015 to January 2021, 678 children aged ≤ 24months who visited Kaohsiung Veterans General Hospital (KSVGH) and had paired urinalysis and urine culture were retrospectively screened. Children with pyuria and positive cultures were classified as having UTI. Pyuria was assessed through urinalysis and defined as a urine white blood cell count (WBC) of ≥5 cells per high-power field (HPF); a positive urine culture was defined as ≥100,000 colony-forming units (CFUs)/ml of a uropathogen for specimens collected using a urine bag or midstream urine. UTIs were diagnosed in 544 children, and 134 children did not meet the diagnostic criteria. Our study was approved by the Institutional Review Board of KSVGH (KSVGH21-CT5-11).

### Study protocol and definitions

The medical records of all children were reviewed, and symptoms relevant to UTI were recorded. Urethral discharge was defined as abnormal purulent or mucoid or greenish secretions seen on the diaper or genital area by caregivers or physicians. Gross hematuria was defined as reddish urine on the diaper and a urine red blood cell count (RBC) of >5 cells/HPF. Malodorous urine was recorded if it was reported by the children’s caregivers. Fever was defined as a core body temperature of >38.0°C. The number of days the child had a fever before receiving treatment was defined by a 24-hour interval rather than caregivers’ reports. The number of days before defervescence was defined as the time between the last day of fever and the time at which antibiotics were first administered. Children were defined to have a fever peak of >39.0°C if a fever of >39.0°C was recorded during hospitalization or by caregivers.

### Variables for comparison

The demographic data, clinical symptoms, fever duration and peak, and urinalysis results of all participants were collected. Data on fever duration before defervescence and urine culture were recorded and analyzed.

### Statistical analysis

All statistical analyses were performed using SPSS for Windows, version 20 (SPSS Inc, Chicago, IL, USA). The diagnostic accuracy of variables was expressed using sensitivity and specificity. Dichotomous variables were compared using the chi-square test or Fisher’s exact test. Continuous variables are expressed as means ± standard deviation and were compared using an independent samples *t* test. Univariate regression was performed, and variables with *P *< 0.1 were further assessed for their association with various factors using logistic regression. The results of the comparison are expressed using *P* values. The statistical significance was set to *P *< 0.05.

## Results

### Ability of urethral discharge to predict UTI

**Figure 1:**
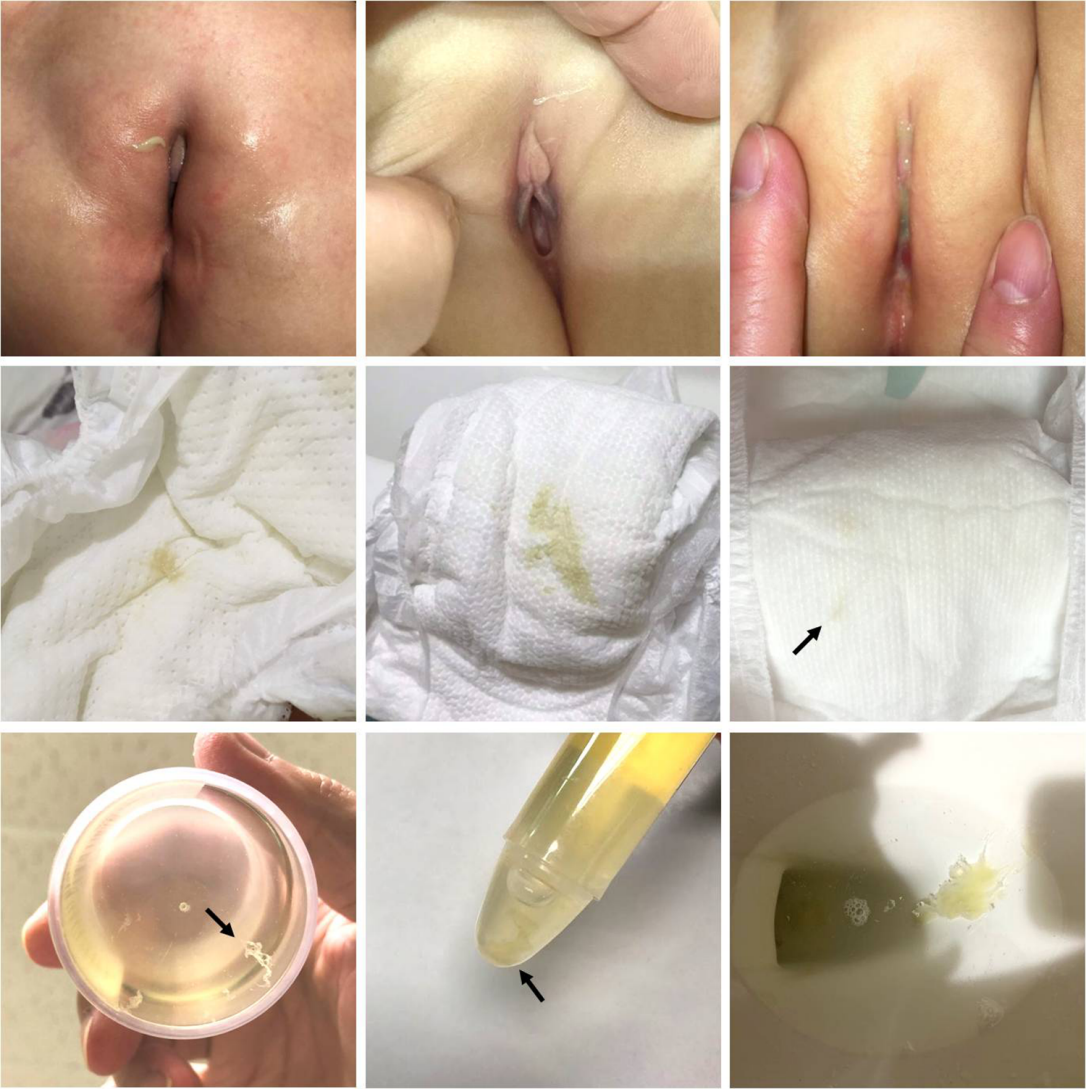
Urethral discharge in children with urinary tract infection. Upper row: greenish and purulent discharge in the vulva area. Middle row: unabsorbed secretion on the diaper, which may require careful inspection to detect (arrow). Bottom row: urethral discharge noted in urine specimen container (arrow) and in toilets.

**Figure 2:**
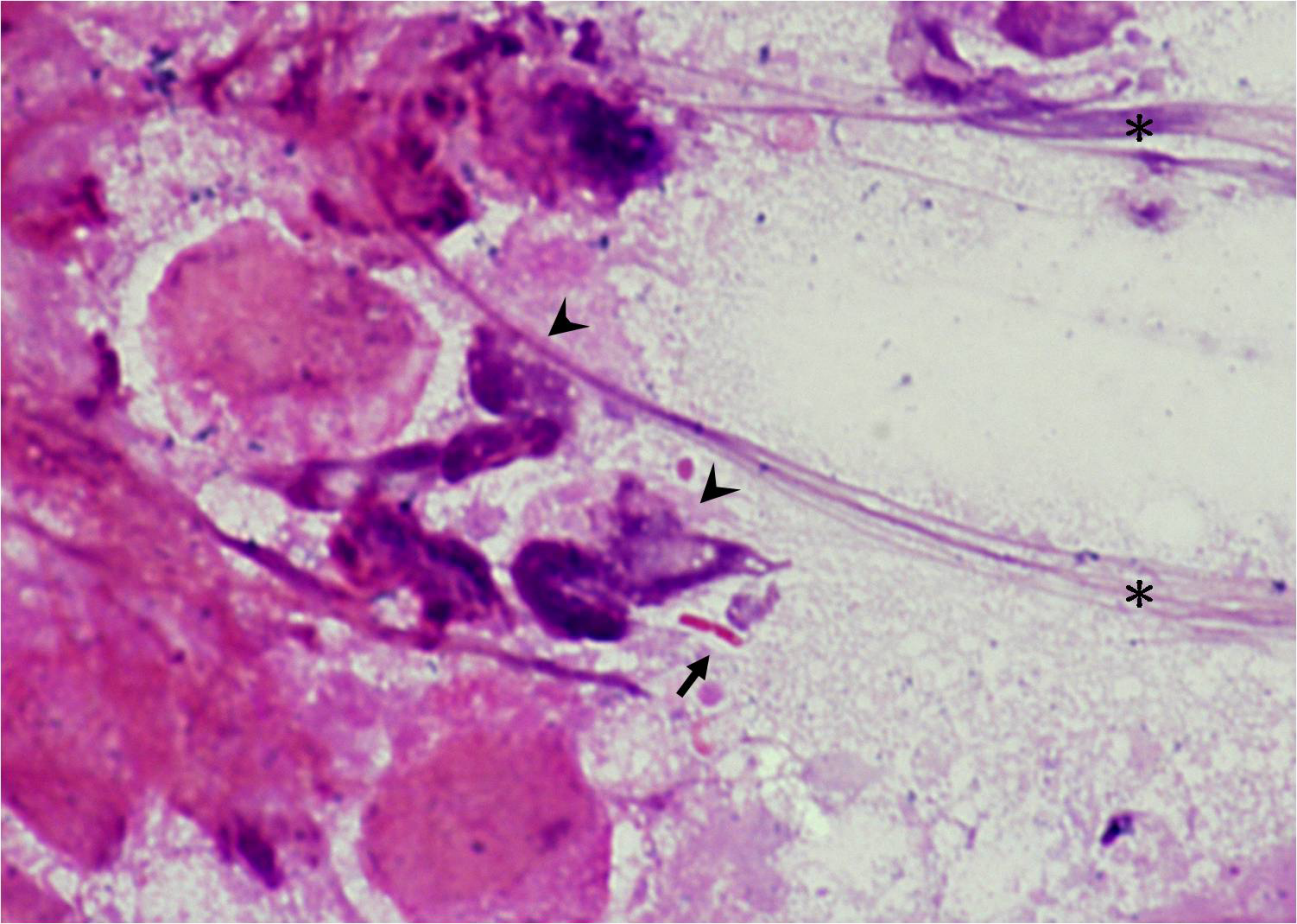
Gram stain of urethral discharge. The Gram stain of urethral discharge revealed Gram-negative *Bacillus*(Arrow) and leukocytes (Arrowhead); a large amount of mucus is noted on the right side (Asterisk).

Among the 678 children in our cohort, 544 were diagnosed with UTI, and 134 did not fulfill the diagnostic criteria. Thirty-eight children presented with urethral discharge, and among them, 28 were diagnosed with UTI. The urethral discharge mostly presented as yellowish or greenish sticky pus on the diaper or genital area. It could be a faint stain on the diaper, making it difficult to observe, and sometimes, the discharge was observed on wiping tissue during a diaper change (Figure 1). Gram stains of urethral discharge revealed mucus-like substances with some leukocytes and bacteria, implying an inflammation process related to bacteria (Figure 2). The sensitivity and specificity of urethral discharge for predicting UTI were 5.1% and 92.5%, respectively, and the positive and negative predictive values of urethral discharge for predicting UTI were 73.7% and 19.4%, respectively.

### Children with UTI presenting with and without urethral discharge

Among 544 children with UTI, 28(5.1%) presented with urethral discharge. The mean age of the cohort was 5.8 ± 5.0 months old, 342(63%) of whom were male. The two groups were comparable in body height and weight (Table 1).

**Table 1:** Characteristics of UTI children presenting with and without urethral discharge.

Variables	Total *n* = 544 (%)	UTI with urethral discharge *n* = 28 (%)	UTI without urethral discharge *n* = 516 (%)	*P*-value
Age, months, mean(±SD)	5.8 (±5.0)	5.6 (±3.7)	5.8 (±5.1)	0.809
Male, *n* (%)	342 (63)	13 (46)	329 (64)	0.065
Body height, mean(±SD)	65.4 (±9.1)	65.6 (±7.4)	65.4 (±9.2)	0.917
Body weight, mean(±SD)	7.4 (±2.1)	7.7 (±1.7)	7.4 (±2.1)	0.578
Clinical manifestations				
Bacteremia, *n* (%)	12 (2)	0 (0)	12 (2)	1.000
Vomit, *n* (%)	69 (13)	6 (21)	63 (12)	0.150
Foul odor urine, *n* (%)	81 (15)	14 (50)	67 (13)	<0.001
Gross hematuria, *n* (%)	20 (4)	3 (11)	17 (3)	0.077
Fever days before treatment (day), mean(±SD)	1.5 (±1.3)	1 (±1.4)	1.5 (±1.3)	0.028
Days before defervescence (day), mean(±SD)	1.3 (±1.2)	0.93 (±1.1)	1.3 (±1.2)	0.084
Received treatment before fever, *n* (%)	28 (5)	9 (32)	19 (4)	<0.001
Fever > 39°C, *n* (%)	298 (55)	5 (18)	293 (57)	<0.001
Urine				
Positive nitrite, *n* (%)	213 (39)	11 (39)	202 (39)	0.988
Leukocyte esterase				1.000
Negative, *n* (%)	12 (2)	0 (0)	12 (2)	
1∼2+, *n* (%)	77 (14)	4 (14)	73 (14)	
3+, *n* (%)	455 (84)	24 (86)	431 (84)	
Gram stain				0.132
Negative, *n* (%)	58 (11)	6 (2)	52 (10)	
Few, *n* (%)	224 (41)	9 (32)	215 (42)	
Many, *n* (%)	262 (48)	13 (46)	249 (48)	
RBC (/hpf)				0.913
<5, *n* (%)	340 (63)	18 (64)	322 (62)	
5–49, *n* (%)	180 (33)	9 (32)	171 (33)	
50–99, *n* (%)	15 (3)	1 (4)	14 (3)	
>100, *n* (%)	9 (2)	0 (0)	9 (2)	
Uropathogen				
*E. Coli*, *n* (%)	482 (89)	19 (68)	463 (90)	0.002
*K. pneumoniae*, *n* (%)	24 (4)	5 (18)	19 (4)	0.005
*Proteus spp*, *n* (%)	16 (3)	2 (7)	14 (3)	0.197
*ESBL E.Coli*, *n* (%)	194 (36)	9 (32)	185 (36)	0.534

ESBL, extended spectrum beta lactamase; HPF, high power field; RBC, red blood cell; SD, standard deviation; UTI, urinary tract infection.

Children with urethral discharge were more likely to present with malodorous urine than those without urethral discharge (50% and 13%, respectively, *P *< 0.001); they were also more likely to have gross hematuria, although this association was not statistically significant (11% and 3%, respectively; *P* = 0.077). No differences were observed in the rate of vomiting or bacteremia between the two groups. In male children, no difference was noted in the prevalence of phimosis in those with and without urethral discharge (69% and 88%; *P* = 0.116). In female children, labial adhesion was also not different in those with and without urethral discharge (0% and 7.4%; *P* = 0.605).

Children with urethral discharge had a lower rate of high fever than those without urethral discharge (18% and 57%, respectively; *P *< 0.001), and the number of days children had fevers before receiving treatment was lower. Nine children (32%) who presented with urethral discharge received antibiotics before fever onset; seven did not develop a fever during the entire UTI treatment course; and two developed a fever after treatment, but defervescence occurred within 1 day. Nonetheless, the days before defervescence after treatment initiation were similar between the two groups (0.93 and 1.3 days, respectively, *P* = 0.084).

Overall, 98% and 89% of children with UTI were positive for leukocyte esterase and bacteria in urinalysis, and the rate was similar in the two groups. Thirty-nine percent of children with UTI were positive for nitrite, and 38% were positive for urine RBC; no differences were observed in these rates between the two groups.

### Uropathogens in children with UTI presenting with urethral discharge

In total, 482 (89%) of UTIs were caused by *Escherichia coli*; however, *E. coli* was less prevalent in children with urethral discharge compared with those without urethral discharge (68% and 90%, respectively; *P* = 0.002). A higher rateof *Klebsiella pneumonia* infection was observed in children with urethral discharge than in those without urethral discharge. No difference was observed in the rate of *Proteus spp.* or ESBL-producing *E. Coli* between the two groups.

### Clinical variables associated with UTI children with urethral discharge

Multivariate forward logistic regression revealed that malodorous urine, infection with *K. pneumonia*, and receiving treatment before fever onset were the factors that were most strongly associated with UTI presenting urethral discharge (adjusted odds ratios [aORs]: 4.79 (1.98–11.6), 9.73 (2.64–35.84), and 9.19 (3.1–27.2), respectively). A fever that spiked above 39 °C and male gender were less strongly associated with UTI with urethral discharge (Table 2).

**Table 2:** Clinical variables related to UTI in children presenting with urethral discharge.

Variables	OR (95% CI)	*p*-value	aOR (95% CI)	*P*-value
Age, months	0.99 (0.92–1.07)	0.808		
Male	0.49 (0.23–1.06)	0.069	0.37 (0.15–0.90)	0.028
Body height	1.00 (0.96–1.05)	0.917		
Body weight	1.05 (0.88–1.26)	0.578		
Clinical manifestations				
Bacteremia	0.71 (0.04–12.26)*	0.813		
Vomit	1.96 (0.77–5.02)	0.160		
Foul odor urine	6.70 (3.06–14.68)	<0.001	4.79 (1.98–11.6)	0.001
Gross hematuria	3.52 (0.97–12.81)	0.056		
Fever days before treatment	0.46 (0.24–0.90)	0.024		
Days before defervescence	0.78 (0.59–1.03)	0.084		
Received treatment before fever	12.39 (4.96–31.00)	<0.001	9.19 (3.10–27.20)	<0.001
Fever > 39°C	0.17 (0.06–0.44)	<0.001	0.23 (0.08–0.70)	0.009
Urine				
Positive nitrite	1.01 (0.46–2.19)	0.988		
Leukocyte esterase				
Negative				
1–2+	1.01 (0.34–3.00)	0.984		
3+	1.18 (0.40–3.50)	0.761		
Gram stain				
Negative				
Few	0.66 (0.29–1.46)	0.322		
Many	0.93 (0.43–1.99)	0.851		
RBC (/hpf)				
<5				
5–49	0.96 (0.42–2.16)	0.913		
50–99	1.33 (0.17–10.48)	0.788		
>100	0.94 (0.05–16.5)*	0.965		
Uropathogen				
*E. Coli*	0.24 (0.10–0.56)	0.001		
*K. pneumoniae*	5.69 (1.95–16.58)	0.001	9.73 (2.64–35.84)	0.001
*Proteus spp*	2.76 (0.60–12.78)	0.195		
*ESBL E.Coli*		0.534		

* Adjusted with Haldane correction.

aOR, adjusted odds ratio; ESBL, extended spectrum beta lactamase; HPF, high power field; OR, odds ratio; RBC, red blood cell, SD, standard deviation; UTI, urinary tract infection.

### Urine characteristic associated with urethral discharge

Urine biochemistry was available for 672 children, 37 of whom presented with urethral discharge. The urine specific gravity, protein, sugar, ketone bodies, occult blood were comparable in both groups, but a higher urine PH value was observed in children with urethral discharge. No difference in the positive nitrite rate or the leukocyte esterase rate was observed (Table 3).

**Table 3:** Urinalysis of children presenting with and without urethral discharge.

Variables	Total *n* = 672(%)	With urethral discharge *n* = 37 (%)	Without urethral discharge *n* = 635 (%)	*P*-value
Specific gravity ≤ 1.015	606 (90)	35 (95)	571 (90)	0.568
pH ≧ 7.0	202 (30)	20 (54)	182 (29)	0.001
Positive protein	407 (61)	19 (51)	388 (61)	0.299
Positive sugar	24 (4)	0 (0)	24 (4)	0.636
Positive ketone bodies	86 (13)	1 (3)	85 (13)	0.073
Positive occult blood	440 (66)	22 (60)	418 (66)	0.478
Positive nitrite	226 (34)	11 (30)	215 (34)	0.721
Positive leukocyte esterase	624 (93)	35(95)	589(93)	1.000

## Discussion

Our study demonstrated that urethral discharge is an uncommon symptom in children younger than 24 months with UTI, but it is highly specific for UTI diagnosis. Urethral discharge may be observed before a fever develops in children with UTI; therefore, the detection of urethral discharge and prompt antibiotic intervention may prevent or alleviate subsequent systemic involvement in children with UTI and, most importantly, prevent kidney scarring due to acute pyelonephritis in the long term.

Fever without other infectious focus was the main symptom in children with UTI, especially those with high spiking fever >39°C (9). The symptoms of UTI are ambiguous and nonspecific in neonates and infants; they include lethargy, vomiting, and prolonged jaundice (8). Gross hematuria, frequent urination, voiding pain, abdominal discomfort, and flank pain are more often described in older children. To prevent kidney damage, early intervention is crucial; it was reported that antibiotics administration after a fever had been present for 24–48 h, 48–72 h, and >72 h resulted in new kidney scars in 5%, 8%, and 14% of children with UTI, respectively (7). However, febrile UTI hints the inflammation of kidney, and infant and young children are often unable to express their discomforts related to UTI, it is therefore difficult for physicians to diagnose UTI in young children without fever because the vague symptoms often do not warrant urinalysis, that is why we need to observe for certain urinary symptoms that warrant urinalysis in these age of group.

Historically, malodorous urine and gross hematuria were symptoms related to young children with UTI. Gauthier et al. recruited 331 children aged 1–36 months in the emergency department. Malodorous urine was reported using parental questionnaires; the incidence was 35.6% in 331 children and 57% in 51 children with UTI. Malodorous urine was related to UTI, with an odds ratio of 2.83 (95% CI: 1.41–5.61) (10). Nonetheless, the authors concluded that the sensitivity and specificity of malodorous urine were too low to diagnose UTI. In addition, all of the children with UTI in the cohort presented with fever, 49% of them had fever ≥ 72 h, malodorous urine was not able to warrant an early diagnosis UTI.

In our study, urethral discharge was considered an early symptom of UTI in young children because it was presented in this population and ceased rapidly after antibiotics administration. In addition, the Gram stain of the discharge revealed leukocytes and bacteria, indicating an infection. Ten children with urethral discharge did not fulfill our diagnostic criteria for UTI, these children may suffered from balanoposthitis, idiopathic urethritis or vaginitis, caused by hormonal imbalance, dysfunctional voiding syndrome, soap or laundry detergents related irritations (11, 12). The incidence of urethral discharge was only 5.1% in children with UTI, possibly because urethral discharge may be difficult to detect; it may appear as a faint stain on the diaper or scant discharge on the vulva or penis of a young infant. Another reason for the low incidence may be the lack of awareness of this symptom in UTI by physicians and caregivers; therefore, a structured questionnaire regarding symptoms in children with UTI may be helpful in the detection of subtle symptoms.

The presence of urethral discharge may be associated with *K. pneumonia* and alkalotic urine. It may be attributed to the urease secreted by *K. pneumonia*, which hydrolyzes urea in the urine to ammonia and carbamate and can thus result in a local rise in urine pH (13). In addition, alkalotic urine precipitates normally soluble polyvalent ions to struvite and carbonated apatite, which may form a stone around the bacterium (14). The Gram stain of urethral discharge in our study did not reveal any stone-like crystals; however, it was difficult to obtain these specimens because of the rare and transient appearance of urethral discharge. Further investigation of urethral discharge may clarify its association with *K. pneumonia* and alkalotic urine.

Our study has several limitations. First, retrospectively reviewing the symptoms of children with UTI may underestimate the true incidence of urethral discharge. Second, because the urine specimens in our study were obtained primarily using a urine bag, positive urine culture results may result from contamination rather than true infection; however, the proportion of false positive UTI should be equally distributed in children with and without urethral discharge, therefore the associations described in our study may still be true.

## Conclusion

Urethral discharge may be an early symptom in children with UTIs, and it may present before fever onset. It is more prevalent in children with alkalotic urine and those infected with *K. pneumonia*. The recognition of urethral discharge as a symptom of UTI may facilitate early intervention and prevent kidney damage.

## Data Availability

The raw data supporting the conclusions of this article will be made available by the authors, without undue reservation.
