# Corrigendum: Urethral discharge as an early manifestation of urinary tract infection in children ≤24 months old

**DOI:** 10.3389/fped.2023.1297186

**Published:** 2023-10-10

**Authors:** Nai-Wen Fang, Shih-Hsiang Ou, Yu-Shan Huang, Yee-Hsuan Chiou

**Affiliations:** ^1^ Department of Pediatrics, Pingtung Veterans General Hospital, Pingtung, Taiwan; ^2^ Division of Pediatric Nephrology, Department of Pediatrics, Kaohsiung Veterans General Hospital, Kaohsiung, Taiwan; ^3^ Division of Nephrology, Department of Internal Medicine, Kaohsiung Veterans General Hospital, Kaohsiung, Taiwan

**Keywords:** urinary tract infection, urethral discharge, gross hematuria, malodorous urine, klebsiella pneuminiae

A Corrigendum on Urethral discharge as an early manifestation of urinary tract infection in children ≤24 months old By Fang N-W, Ou S-H, Huang Y-S and Chiou Y-H (2023) Urethral discharge as an early manifestation of urinary tract infection in children ≤24 months old. Front. Pediatr. 11:1149218. doi: 10.3389/fped.2023.1149218

In the published article, there was an error in affiliations 1 and 2. Instead of “Division of Pediatric Nephrology, Department of Pediatrics, Kaohsiung Veterans General Hospital, Kaohsiung, Taiwan”, it should be “Department of Pediatrics, Pingtung Veterans General Hospital, Pingtung, Taiwan”. Instead of “Department of Pediatrics, Pingtung Veterans General Hospital, Pingtung, Taiwan”, it should be “Division of Pediatric Nephrology, Department of Pediatrics, Kaohsiung Veterans General Hospital, Kaohsiung, Taiwan”.

As the affiliation order changes, authors Yu-Shan Huang and Yee-Hsuan Chiou should be affiliated with affiliation 2.

The authors apologize for this error and state that this does not change the scientific conclusions of the article in any way. The original article has been updated.

